# Perturbations of Lipid Metabolism Indexed by Lipidomic Biomarkers

**DOI:** 10.3390/metabo2010001

**Published:** 2012-01-04

**Authors:** Antonin Lamaziere, Claude Wolf, Peter J. Quinn

**Affiliations:** 1 INSERM U1057, Faculte de Medicine, “P. et M. Curie” 27 rue Chaligny, Paris 75012, France; Email: antonin.lamaziere@upmc.fr; 2 Department of Biochemistry, King’s College London, 150 Stamford Street, London SE1 9NH, UK; Email: p.quinn@kcl.ac.uk; 3 Laboratory of Mass Spectrometry, Applied Lipid Investigation (APLIPID®), Paris 75012, France

**Keywords:** biomedical lipidomics, pharmacology, biomarkers

## Abstract

The lipidome of the liver and the secreted circulating lipoproteins can now be interrogated conveniently by automated mass spectrometric methods. Multivariate analysis of the liver and serum lipid composition in various animal modes or in human patients has pointed to specific molecular species markers. The perturbations of lipid metabolism can be categorized on the basis of three basic pathological mechanisms: (1) an accelerated rate of *de novo* lipogenesis; (2) perturbation of the peroxisome pathway of ether-lipid and very-long-chain fatty acid biosynthesis; (3) a change in the rate of interconversion of essential omega-3 and -6 polyunsaturated fatty acids. This review provides examples to illustrate the practicalities of lipidomic studies in biomedicine.

## 1. Introduction

Biological tissues are comprised of different proteins, lipids and carbohydrates in varieties and relative proportions that are required to sustain the functional living state. Proteins are direct products of the genetic apparatus and regulation of their synthesis and functions are largely understood. Likewise, the carbohydrates whilst representing products a step removed from genetic origin fulfill metabolic and structural roles that appear relatively straightforward. The lipids, by contrast, are of a different order of chemical complexity that defies definition beyond the shared level of their solubility in solvents of low polarity. Moreover, the underlying reasons for the biochemical diversity represented by hundreds of molecular species, which is observed within each of the major lipid classes, have so far to be explained.

The methods of choice for analysis of lipid extracts nowadays are the, so-called, soft ionization methods of electrospray ionization coupled with single stage or tandem mass spectrometry. A method, referred to as shotgun lipidomics, when applied to the infusion of whole tissue lipid extracts, generates molecular ions without extensive fragmentation of the molecules. It allows the identification and quantitation of the lipids in a mixture and facilitates the high throughput global analysis of a cellular lipidome [1,2]. The procedures generate quasi-molecular ions in high yields. Their cleavage by collision with gas molecules in tandem equipment or by low energy excitation in ion trap has greatly facilitated assignment of structures. Implementing fragmentation of parent lipid ions and high mass resolution filtering in the mass spectral equipment available for clinical laboratories explains the development of lipidomics for clinical studies. This development in conjunction with more sophisticated sample handling methods, data reduction and analysis now means that the entire lipidome can be revealed rapidly in medical samples of ever diminishing size.

While considerable advances have been made in the study of protein and carbohydrate functions in biological organisms through the development of proteomic and glycobiological techniques, analytical methods applied to the lipidome are only now coming into the fore for their interest in biomedicine. Not only do these research oriented lipidomic studies enable the characterization and quantitative determination of the full panoply of lipid molecular species in serum and liver extracts the assessment can now be undertaken with impressively small samples available for clinical investigations. Apart from improvements in spectrometric techniques the major challenges remaining in the lipidome field are in the processing, analysis and presentation of the enormous volume of data generated by contemporary mass spectrometric analysis. This is an essential step in unraveling the factors responsible for regulating lipid metabolism and ultimately is key to their use for clinical medicine.

In this review we use examples of how mass spectrometric approaches to analysis of the lipidome can be used to detect perturbations of lipid metabolism associated with specific pathological states in human and model animals prepared for drug testing. We will focus attention only on lipids of the liver and serum lipoproteins. The utility of the method will be discussed in the context of its biomedical applications.

## 2. The Integrated Lipidome Knowledge Base

Most pathways of lipid metabolism are well known. Where greater understanding is required is how these pathways operate in integrated biological systems to support individual metabolic health and, importantly, how changes in the regulation of these pathways can lead to major metabolic diseases. Indeed most human diseases are complex multi-factorial states resulting from a combination of various genetic and environmental factors. Fortuitously for lipidologists, the analytical tools required to consolidate this integrated knowledge are emerging. For the first time researchers can now measure vast portions of the lipid metabolome and, with access to appropriate analytical procedures, can produce accurate quantitative measurements.

A meaningful way to interpret the variations in the lipidome requires the integration of lipid as well as non-lipid (*i.e.*, transcriptome and genome) parameters. The KEGG (Kyoto Encyclopedia of Genes and Genomes) pathway data bank is a useful database to start from. The database contains a compendium of manually drawn graphical diagrams, referred to as KEGG pathway maps, representing molecular pathways for metabolism, genetic information processing, environmental information processing, other cellular processes, human diseases, and drug development (http://www.genome.jp/kegg/) [3]. The KEGG pathway database contains pathway maps in both normal and perturbed states. Diseases are regarded in the database as perturbed states of the molecular system and drugs as acting to perturb the system. Disease information is computerized in two forms: Pathway maps and gene/molecule lists. The KEGG drug database contains chemical structures and/or chemical components of all Japanese drugs, traditional chinese medicine formulas, and drugs available in the USA and Europe. This database also incorporates information about two types of molecular networks: The interaction network with target molecules, metabolizing enzymes, other drugs, *etc.* and the chemical structure transformation network in the history of drug development. The new disease/drug information resource referred to as KEGG medicus can be used as a reference knowledge base for computational analysis of lipidome, especially.

## 3. Development of Lipidomics for Biomedical Applications

Fatty acid profiling originally based on gas chromatographic analysis has gradually been replaced by mass spectrometry-based lipidomics in the clinical biochemical situation. The rationale to seek biomarkers at the level of an individual lipid molecular species is to improve diagnostic confidence and sensitivity in a range of clinical conditions [4]. A so-called “lipid analysis” is already a consistent component of blood tests routinely prescribed by physicians. The follow up of treatment by drugs that alter the levels of major plasma lipids such as cholesterol and triglycerides is also calibrated for these simple assays. Although plasma contains many thousands of distinct lipid molecular species six main categories are recognized including fatty acyls, glycerolipids (neutral), glycerophospholipids (polar), sphingolipids, sterols, and prenols. How the individual molecular species comprised in these lipids contribute to the normal physiological processes of the body and how their levels change in response to therapy has remained largely obscure until MS-based lipidomic studies reveal their diversity.

As we learn more about the specific actions of individual lipids in health and disease, it may be anticipated that blood tests in future will demand the inclusion of an ever increasing number of lipid molecules. Inevitably this means that screening of lipid biomarkers will be the next step and the resource of lipidomics will become practical if only a few molecular species markers serve to alert the clinician. Currently, the elevated cost of many comprehensive lipidomic studies is supported for drug development and public health such as in the large-scale nutrition surveys. Lipidomics has yet to be applied for personalized medicine. Before this can be accomplished laboratory facilities and instrumentation will need to be established for automated analysis and interpretation which requires sophisticated statistical processing of large data files until the screening identifies the relevant lipid biomarkers. An essential component of this intermediary step in the development of the method is cooperation between industries, research agencies and biotech companies specialized in lipidomics. It is now five years since it was pointed out by Wiest [5] that “Lipidomics technologies do not guarantee a fast track to new knowledge. The vast amount of data produced by these platforms presents a major hurdle to assembling valid knowledge and to the discovery of mechanistic biomarkers.” It seems that we have journeyed only part of this long track up to now.

## 4. Why Serum Analysis Can Serve for Investigations of Liver Function

**Figure 1:**
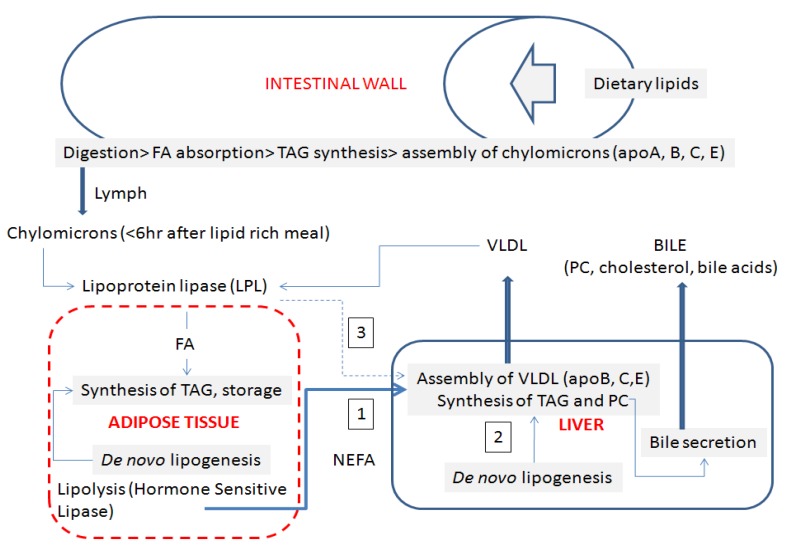
Pathways of lipid metabolism and traffic in liver.

The accretion of lipids in the liver is dependent on the balance struck between lipid synthesis (lipogenesis), lipid export to the serum as lipoprotein (VLDL), and bile secretion highly enriched with sterols and lecithin. A scheme outlining lipid metabolism in liver is presented in Figure 1. Triglyceride (TAG) synthesis in the liver utilizes fatty acids (FA) originating from 3 distinct sources as a substrate.

Non esterified fatty acids (NEFA) are released in the serum by the hormone-sensitive lipase (HSL) activated in the fasting state (arrow 1). The liver takes up the products of this adipose lipolysis when FA are not diverted by muscles for sustaining a prolonged exercise. *De novo* synthesis of fatty acids from acetyl-CoA (arrow 2) is activated by insulin when the liver supply of glucose exceeds the need for restoration of the liver glycogen dependent on insulin in the fed state. In the feed state, fatty acids (FA) are also released by the lipoprotein lipase activity (LPL) bound at the luminal surface of endothelial cells of capillaries irrigating the adipose tissue. From the capillaries a fraction of the FA is released from the LPL substrates, chylomicrons and VLDL, which escapes (“spill over”) the uptake by adipocytes and is diverted to the liver (arrow 3). In 9 obese patients affected by a severe insulin resistance and “fatty liver” it has been estimated that the ratio of pathway 1:pathway 2:pathway 3 is around 3:1:0.5 [6].The ratio would have been different in healthy insulin responsive subjects because pathway 1 is shut down in the fed state by insulin. Lipogenesis rate is normally balanced such as no “fatty liver” occurs. However the high insulin levels that compensate for the decreased insulin resistance accelerate *de novo* lipogenesis from acetyl-CoA (arrow 2) and can result in liver steatosis.

In addition to VLDL secretion a portion of the fatty acid influx to the liver is balanced by the abundant biliary efflux of lecithin (noticeably the bile phosphatidylcholine is comprised of *sn*-1-palmitoyl-*sn*-2-linoleyl- molecular species). The biliary efflux is rapidly compromised by cholestasis [7]. Because of the precise balance of FA influx *versus* triglyceride, cholesterol and phospholipid efflux and the insulin/adipocytokines regulation of *de novo* lipogenesis, no lipid storage is normally observed in the liver. By contrast, the adipose tissue can develop a wide hypertrophy for storage.

The scheme shown in Figure 1 explains why biliary blockade (*i.e.*, cholestasis) as well as decreased VLDL secretion into the serum (see paragraph “Serum VLDL reflects a deficit of phosphatidylcholine synthesis in liver”) are causes leading to fatty liver. This shift in lipid traffic is rapidly manifest because the liver synthesizes large daily amounts of lipids that are normally exported to peripheral tissues but not stored *in situ*. By contrast, the adipose tissue behaves as an expendable compartment. Lipotoxicity can only be observed for large excess of adipose tissue. Liver lipotoxicity is also correlated with high levels of circulating NEFA which “activate the intrinsic apoptosis pathway in hepatocytes via c-Jun *N*-terminal kinase (JNK) [8,9]. JNK activates the proapoptotic protein Bim, resulting in Bax activation and enhanced apoptosis, termed ‘lipoapoptosis’ [10]. Reduced adiponectin levels may establish also a proinflammatory milieu, thus increasing vulnerability to lipotoxicity, which promotes progression from simple steatosis to non-alcoholic steatohepatitis (NASH) and even advanced hepatic fibrosis” [11]. As indicated below in Section 7 the progression of lipotoxicity can be followed stepwise by detailed lipidomic investigation of the serum [12].

## 5. Clinical Investigations of Fatty Liver

Until recently clinicians investigating fatty liver conditions did not regard the sophisticated lipidomic methods set up by biochemists as particularly relevant. Imaging of fatty liver by ultrasound examinations was thought sufficient to complement the routine biochemical blood tests in individual or epidemiologic studies of fatty liver [13]. In a 2008 review liver biopsy was considered the gold standard for detecting and staging fatty liver disease as steatosis alone (Non-alcoholic fatty liver disease: NAFLD), which has a benign course and steatohepatitis or non-alcoholic steatohepatitis (NASH), which may be associated with fibrosis, and progression to cirrhosis and hepatocellular carcinoma (HCC) [14]. Classically, patients with NAFLD have slightly elevated liver enzyme values, deny excessive alcohol consumption, and have negative serological tests for viral hepatitis, autoimmune liver disease and congenital causes of chronic hepatitis, information allowing a negative differential diagnosis more than a positive diagnostic. Bellentani *et al*. [15] have found ultrasound evidence of fatty liver in 46% of non-obese and 95% of obese heavy drinkers, demonstrating that obesity doubles the prevalence of alcohol-induced fatty liver disease. However the appreciation and the threshold for detection of fatty liver, which remains relatively inaccurate by liver echography, should be revised since Westernized societies are becoming conscious that they are facing an epidemic proportion of NAFLD patients. Prevention on a population scale with only lifestyle adjustments, such as dietary adaptation and exercise, will most likely require an accurate monitoring protocol of greater sensitivity and more applicability than ultrasound examination. Thus lipidomics is expected to provide a novel toolkit to unravel the relationships between hypercaloric diets and other environmental and genetic factors associated with the metabolic syndrome [16].

The advent of lipidomics in a biomedical context has made an appearance long after the establishment of routine liver biochemical tests. For many clinical situations, however, it is necessary to challenge a lipidomic approach against tried and tested biochemical methods. For an example, many tests for cholestasis such as total cholesterol, total bile acid, bilirubin and serum level of various enzymes (alkaline phosphatases, 5′-nucleotidase *etc.*) exported into the bile are often judged to be sufficiently sensitive to detect the reflux of metabolites over the biliary blockade encountered in most clinical situations. However, it is acknowledged that much greater sensitivity may be required to detect the early subtle alterations such as occur in gravidum cholestasis. Thus detection and interpretation of circulating lipids and bile acids by lipidomics methods at an early stage can lead to timely management in a clinical context and may reduce complications in both mother and fetus [17].

Other examples of where lipidomic analysis can improve diagnosis of liver lipid metabolic disorders will now be considered.

### 5.1. Serum Glycerides

The measurement by routine assay methods of the level of circulating total glycerides is usually judged appropriate for the clinical assessment of lipid metabolism. Indeed, glycerides are comprised of triglycerides summed with diglycerides, which are assayed jointly in the automated glycerol assay method. This approximation is not a drawback for an assessment of dyslipemia for assessing the cardiovascular risk [18]. For this purpose, the distinction of lipoproteins conveying cholesterol in the serum has to be obtained because only LDL infiltrates the arterial intima. HDL and VLDL that is highly enriched in triglycerides are innocuous. Differently it is necessary to revise the total glyceride assay when investigating insulin resistance in the context of metabolic syndrome (MSy) and fatty liver because of a specific significance of diglycerides. Together with ceramides a role of diglycerides is currently debated as a toxic mechanism in the liver [19]. The importance of separating diglycerides and triglycerides is also highlighted for the trial of inhibitors of triglyceride synthesis administered as candidate drugs in the treatment of MSy [20].

### 5.2. Serum VLDL Reflects a Deficit of Phosphatidylcholine Synthesis in Liver

Patients affected by a deficit in lipotropic factors are at a high risk of developing fatty liver. A detailed lipidomic profile of serum phosphatidylcholine is required to monitor the follow up of such a condition. The treatment aims to supplement the patient with lipotropic metabolites such as betaïne, choline and methionine required for liver lecithin synthesis. Lipotropes act as methyl donor substrate for the phosphatidylethanolamine methyl transferase, which synthesizes nearly half of phosphatidylcholine in the liver. The consequences of an insufficient dietary supply of these lipotropic factors on VLDL lipoprotein metabolism have been reviewed [21]. Phosphatidylcholine is the only phospholipid required for lipoprotein assembly and secretion. Impaired hepatic biosynthesis of phosphatidylcholine results in a significant reduction in levels of circulating very low density lipoproteins and high density lipoproteins (HDLs). The reduction in VLDLs appears to be due, in part, to impaired secretion of VLDLs from the liver. Less phosphatidylcholine within the hepatic secretory pathway results in nascent VLDL particles with reduced levels of phosphatidylcholine recognized as being defective and degraded within the secretory system in a post-endoplasmic reticulum compartment. Moreover, VLDL particles are taken up more readily from the circulation when the phosphatidylcholine content of the VLDLs is reduced. This could be due to a preference of cell surface receptors and/or enzymes for lipoproteins that contain less phosphatidylcholine.

Insufficient dietary intake of choline in humans causes a retarded assembly of VLDL by the liver. A portion of the choline requirement is met via endogenous *de novo* synthesis of phosphatidylcholine catalyzed by phosphatidylethanolamine *N*-methyltransferase (PEMT) in the liver. Though many foods contain choline, many humans do not get enough in their diets. When deprived of dietary choline, most adult men and postmenopausal women developed signs of organ dysfunction such as fatty liver and VLDL deficient in phosphatidylcholine. Wide differences in requirement for choline occurring among population subgroups has been recognized [22]. A noteworthy feature is a major gender difference resulting from estrogen levels which induce expression of the PEMT gene and allows premenopausal women to utilize their endogenous choline requirements more efficiently. In addition, there is significant variation in the dietary requirement for choline that can be explained by common polymorphisms in genes involved in choline and folate metabolism.

### 5.3. Measurement of de Novo Lipogenesis from Serum Lipidomics

One of the major advantages of lipidomic analysis relevant to many clinical developments is the possibility for measurement of the rate of lipogenesis (Figure 1; arrow 2). Routine biological tests do not allow such appreciation. Moreover the measurement can be obtained from a serum sample without recourse to a liver biopsy. By means of a simple semi-quantitative lipidomic analysis, an appreciation of fatty acids originating from *de novo* synthesis is given as a ratio to other fatty acids. The ratio can be established over FA with an obligatory dietary source such as essential fatty acids. The ratio can be used, for example, to monitor repeatedly the correction of an accelerated rate of lipogenesis under antidiabetic treatment. On the opposite a quantitative absolute determination of the rate of lipogenesis is not a practical issue in a clinical context because administration of deuterium or ^13^C isotope-labeled fatty acid precursors must be maintained for several days to achieve a steady state concentration. For a research oriented measurement of human lipogenesis using deuterium incorporation, for example, D_2_O must be given orally for 4 days before the turn-over of fatty acids in circulating triglycerides can be calculated [23].

**Figure 2:**
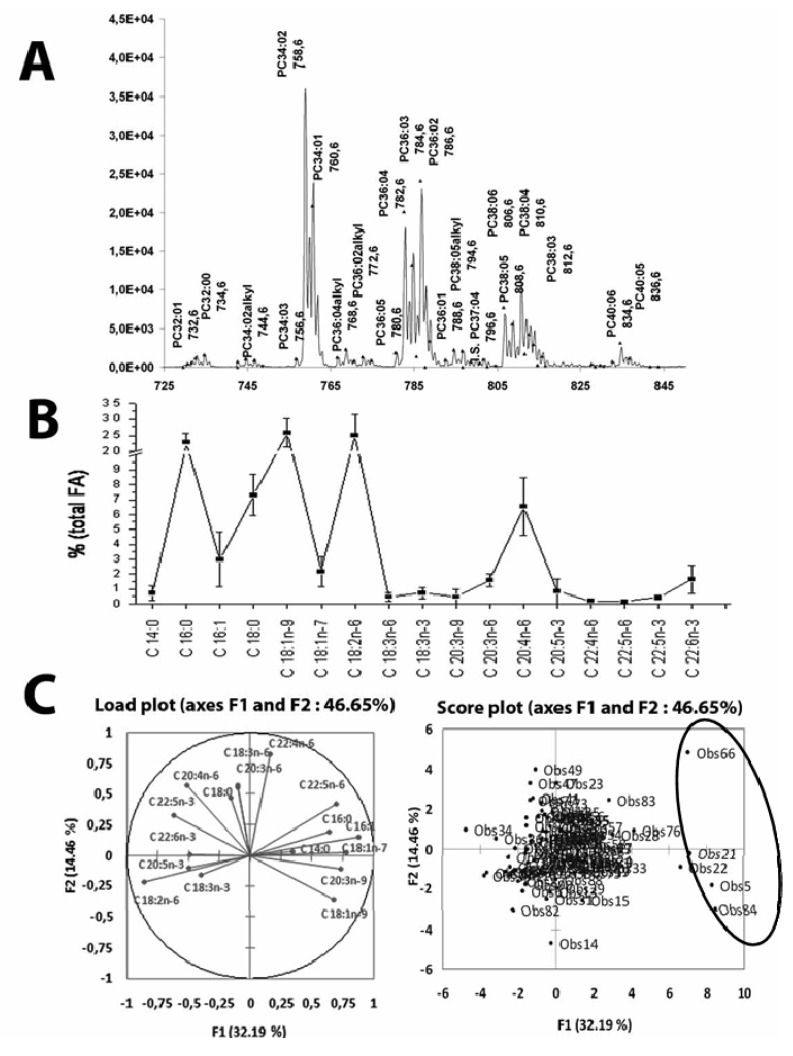
(**A**) Identification of molecular species of phosphatidylcholine from total lipid analysis of serum. (**B**) The distribution of 17 major fatty acids in the total circulating lipidome was obtained by gas chromatography of fatty acid methyl esters prepared by acidic transmethylation. (**C**) Sera from 93 patients (adults and children) were randomly sampled. Four sera (Obs 66, 21, 5 and 84) show an enrichment in fatty acid marking the accelerated *de novo* lipogenesis; fatty acid 16:1 (palmitoleic), 18:1ω7 (vaccenic), 20:3ω9 (mead acid) are increased in these patients scored in left part of the biplot. The enrichment is recorded at the expense of the ω-3 essential fatty acid (C 22:6ω3 (docosahexaenoic), 20:5ω3 (eicosapentaenoic), 22:5ω3 (docosapentaenoic acid)) shown “diametrically” opposite in the right part of the biplot. The biplot is obtained by the superimposition of the load plot for the variations of serum fatty acids (the factorial plan F1/F2 represents 46% of the total variations) and the score plot for the 93 observations.

Lipidomics reveals the multiple molecular species within each class, *i.e.*,the variety of fatty acids comprised in individual lipid molecules within each lipid class. A “simplified profile” derived from mass spectrometry can be readily obtained. This profile is different from the FA composition because mass spectra contain the information as a total carbon and double-bond number in the 2 or 3 acyl chains esterified in the lipid. However, each of the acyl chain structure is not apparent from a simple scan of the parent lipid ions. For instance, a triglyceride quoted as TG 52:3 may be either 16:0/18:0/18:2 or 16:1/18:1/18:0. Similarly PC 34:2 may be either 16:0/18:2 or 16:1/18:1. This is exemplified in Figure 2, which shows a mass spectrum of liver or serum phosphatidylcholine. The peak corresponding to PC 34:2 is enlarged to show the MS2 fatty acid composition.

A clinical appreciation of *de novo* lipogenesis relies on a few fatty acids that serve as specific biomarkers. The complete fatty acid composition need not to be resolved to perform the measurement as only the ratio of a marker of *de novo* lipogenesis over other fatty acids is required. As an example, palmitoleic acid (16:1) can be found associated with a variety of acyl chains combined in lipids. Nevertheless its significance as a marker of an accelerated rate of *de novo* lipogenesis remains similar whatever the particular lipid it is esterified to. In the serum, 3 abundant lipid classes are comprised of palmitoleic acid; triglycerides, phosphatidylcholines and cholesterol esters. Triglycerides and phosphatidylcholines are synthesized in the liver and secreted on lipoproteins into the serum. Cholesterol esters are synthesized in the serum HDL by a transacylation of phosphatidylcholines (lecithin cholesterol acyltransferase, LCAT, is activated by apoA1). Therefore all 3 serum lipids, triglycerides, phosphatidylcholines and cholesterol esters contain the palmitoleyl acyl chain from the same liver source with similar marker significance. Difference in the efficiency of the marker arises from the distinct turn-over of the lipid class (faster for triglycerides than cholesterol esters). Because the measurement does not require a time-consuming determination of each structure comprised in the mass spectrum by MS2 experiments the “profile marker method” is amenable in the context of a clinical laboratory in the form of an index. By contrast, in a research-oriented study, MS2 will have to establish the detailed structure of each mass peak in the mass spectrum by the cleavage/identification of product ions after a collision-induced dissociation of the parent lipid anion. The later procedure can be manually activated. Alternatively a so-called “Intelligent Data Acquisition” module of the acquisition software can also be automated during the chromatography run. The set-up of the conditions activating the module has not proven to be practical when highly variable serum samples from patients are run in series.

**Figure 3:**
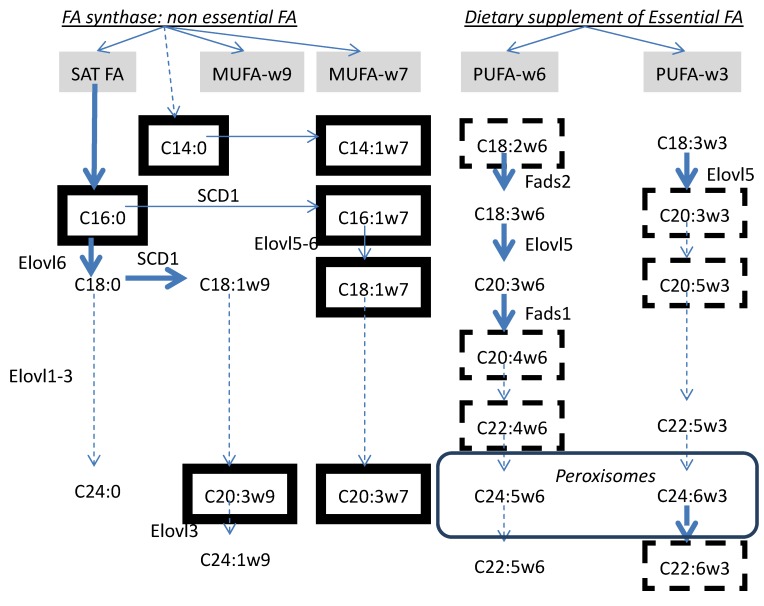
Relationships between endogenous biosynthesis of non-essential fatty acids and dietary sources of essential fatty acids. The separation between pathways leading to the synthesis of non-essential fatty acids (saturated (SAT) and monounsaturated (MUFA-ω7 and -ω9 families) and essential polyunsaturated fatty acids (PUFA-ω6 and -ω3 families) is shown. The interaction (competition for substrate, gene expression) between the separated pathways is presented in the text. Adapted from reference [42].

Profiling by GC of total lipid fatty acid as methyl esters is considered a simple method with the capacity to evaluate the rate of lipogenesis. It can be seen in Figure 2B that in large (n = 93) series of serum from outpatients with different lifestyle and diet the fatty acid profile is maintained in a relatively narrow corridor. This is the result of an accurate homeostatic adjustment of fatty acid metabolism by the liver, *i.e.*, synthesis and interconversion of the fatty acid precursors. Figure 2c shows that Principal Component Analysis (PCA) of the total serum fatty acid profiles can sort out 5 patients with an accelerated rate of lipogenesis. These samples are conspicuously enriched in 16:1 (palmitoleic), 18:1ω7 (vaccenic), 20:3ω9 (mead) and 18:1ω9 (oleic) compared with the average values in the population tested, all 4 fatty acids being products by *de novo* lipogenesis (see Figure 3). The enrichment in 22:5ω6 will be discussed below in the context of essential fatty acid deficit as a “over-compensation” reaction.

#### 5.3.1. Biomarker Selection

Markers for the essential fatty acid (EFA) deficit syndrome are also shown in Figure 3. Indeed, EFA deficit is invariably associated with an increase in *de novo* lipogenesis markers in children with cystic fibrosis. In adults with deteriorated liver condition the association with *de novo* lipogenesis may not be observed. In point of fact, 18:2ω6 (linoleic), the dietary precursor of the ω-6 family, and 22:6ω3 (docosahexaenoic acid, DHEA) are inhibitors of *de novo* lipogenesis and act to repress a cluster of lipogenesis genes expression in the liver [24]. In the particular example shown in Figure 2C, EFA are decreased in the children affected by cystic fibrosis because of the deficit in pancreatic lipase available for the intestinal lipid digestion. In this particular instance, EFA deficit is causal and *de novo* lipogenesis is accelerated secondarily.

The interactions between *de novo* lipogenesis and ω6- and ω3-EFA interconversion may become even more complex as the liver deteriorates. While essential fatty acid precursors linoleic (18:2ω6) and linolenic (18:3ω3) of the ω-6 and ω-3 series, respectively, are not synthesized *de novo* in man, the liver proceeds actively to their interconversion to very long polyunsaturated end-products (Figure 3). Linoleic acid (18:2ω6) is elongated and desaturated to arachidonate (20:4ω6) and EPA (20:5ω3) to DHA (22:6ω3)). The deterioration of peroxysomal activity, where the very long chain synthesis proceeds has served to distinguish benign fatty liver disease and steatohepatitis [25].

#### 5.3.2. Sources of Fatty Acids Stored in Liver and Secreted via VLDL Lipoprotein as Triglycerides

Regulation of lipogenesis changes the amounts of triglyceride that is exported to the serum. The sources of fatty acids stored in liver and secreted via lipoproteins in patients with nonalcoholic fatty liver disease has been established from isotope studies [6]. In these studies the sources of hepatic and plasma lipoprotein triacylglycerides were directly quantified in NAFLD. Patients (5 male and 4 female; 44 ± 10 years of age) were infused with and orally fed stable isotopes for 4 days to label and track serum nonesterified fatty acids (NEFAs), dietary fatty acids, and those derived from the *de novo* lipogenesis (DNL) pathway, present in liver tissue and lipoprotein triacylglyceride. Hepatic samples from the patients scheduled for a medically indicated liver biopsy and lipoprotein triacylglyceride fatty acids were analyzed by gas chromatography/mass spectrometry. NAFLD patients were obese, with fasting hypertriglyceridemia and hyperinsulinemia. Of the triacylglycerol accounted for in liver, 59.0% ± 9.9% of triacylglycerol arose from NEFAs (arrow 1 in Figure 1); 26.1% ± 6.7%, from *de novo* lipogenesis (arrow 2 in FIGure 1); and 14.9% ± 7.0%, from the diet (arrow 3 in Figure 1). The pattern of labeling in VLDL was found to be similar to that in liver, and throughout the 4 days of labeling, the liver demonstrated reciprocal use of adipose and dietary fatty acids. *De novo* lipogenesis was elevated in the fasting state and demonstrated no diurnal variation. These quantitative metabolic data demonstrated that both elevated peripheral fatty acids and *de novo* lipogenesis contribute to the accumulation of hepatic and lipoprotein fat in NAFLD. The study clearly established that circulating high “free fatty acid” level is the origin of an excessive liver lipid accumulation. High NEFA levels are related to obesity and an expanded adipose tissue explaining the FA “spill-over” and the insulin-resistance to activate *de novo* lipogenesis.

Indeed, a wide variety of pathological conditions result in the same lipid perturbations. Lipidomics will provide the means to understand the differences between these conditions under the influence of treatment. Clinical trials using routine lipid parameters have already found differences in insulin resistance and accelerated lipogenesis in metabolic syndrome (MSy), type 2 diabetes (T2D), polycystic ovarian syndrome (POS), non-alcoholic fatty liver (NAFLD), steato-hepatitis (NASH) and ω-3 essential fatty acid deficit [26,27,28,29].

## 6. Hepatitis Virus C Infection Perturbs Lipogenesis

Evidence derived from sensitive lipidomics methods of investigating mechanisms of HVC replication in infected patients can be taken as an example of how a basic understanding of lipogenesis can be translated into a future biomedical application. Though circulating levels of triglycerides may be normal or typically low, accelerated liver lipogenesis is associated with chronic infection by hepatitis virus C. The association of fatty liver and hepatitis C virus infection may be considered circumstantial if judged only by epidemiology. However, hepatitis C virus (HCV) is known to enhance its replication by co-opting the host liver lipid metabolism [30,31]. HCV circulates in the blood embedded in VLDL-like lipoproteins. HCV infection is associated with enhanced lipogenesis but reduced secretion of VLDL [32]. Many lipids are crucial for the virus life cycle, and consequently many inhibitors of cholesterol/fatty acid biosynthetic pathways inhibit virus replication. Components involved in VLDL assembly are also required for HCV morphogenesis/secretion, suggesting that HCV co-opts the VLDL secretory pathway for its own secretion. HCV alters cellular lipid metabolism to create a lipid-rich intracellular environment to facilitate its own multiplication. HCV activates sterol regulatory element binding proteins (SREBPs), the master regulators of cholesterol/fatty acid biosynthesis. HCV core protein in the presence of proteasome activator PA28-gamma activates SREBP1c. HCV down-regulates very-low-density lipoprotein (VLDL) particle secretion by inhibiting the activity of microsomal triglyceride transfer protein (MTP) activity. The rearrangement and aggregation of lipid droplets induced by HCV core protein also interferes with VLDL assembly. These events disturb lipid homeostasis leading to the intracellular accumulation of lipid droplets, and this manifests as steatosis, the prominent pathological phenotype associated with HCV infection.

The mechanism of insulin-resistance in HVC infection has been recently reviewed [33]. Epidemiological studies have shown that chronic hepatitis C induces insulin resistance. In turn, insulin resistance in chronic hepatitis C is associated with progression of liver fibrosis, resistance to antiviral therapy and development of hepatocellular carcinoma. A recent study has revealed that co-infection with HIV and HCV enhances the release of fatty acid synthase into the circulation suggesting the enzyme is targeted specifically by HVC [34]. Serum fatty acid synthase concentration was found higher in HIV-infected patients than in healthy participants and HCV co-infected patients showed higher levels than those without co-infection. Insulin concentration was the sole variable among metabolic parameters that demonstrated a significant correlation with serum fatty acid synthase concentrations suggesting the co-development of insulin-resistance. A simple correlation may be difficult to draw since the activity of the released enzyme is unknown and these patients have also received an antiviral treatment with an effect on the insulin-resistance. The enhanced expression of pro-inflammatory mediators and liver X-receptor-regulated lipogenic genes in non-alcoholic fatty liver disease and hepatitis C were more easily explained [35]. The hepatic expression of LXRα and its lipogenic and inflammatory targets was evaluated in 43 patients with NAFLD, 44 with chronic HCV infection and in 22 with histologically normal liver. LXRα gene and its lipogenic targets PPAR-γ (peroxisome-proliferator-activated receptor-γ), SREBP (sterol-regulatory-element-binding protein)-1c, SREBP-2 and fatty acid synthase were overexpressed in the liver of NAFLD and HCV patients who had steatosis.

## 7. NASH and NAFLD have Distinct Lipidomic Profiles

The combination of lipid perturbations especially those peroxisome pathways for very long (>C22) chain fatty acid synthesis and degradation and ether-phospholipid synthesis (alkyl- and alkenyl-*sn1*-phospholipid) can be superimposed on the biomarkers for insulin resistance and accelerated lipogenesis syndrome. This superimposition is highly informative for a follow-up of the severity of the liver condition and it is already proven to be of diagnostic value to distinguish between NAFLD and NASH. Whereas the staging relies until now on the liver biopsy it would be a significant improvement to follow the conversion of the benign NAFLD to a complicated form on the basis of a serum investigation alone [12]. The plasma lipidome of NAFLD and NASH have a distinct plasma lipidomic signature for plasma lipids and eicosanoid metabolites. The key findings include significantly increased total plasma monounsaturated fatty acids driven by palmitoleic (16:1ω7) and oleic (18:1ω9) acids content (*p* < 0.01 for both acids in both NAFL and NASH). The levels of palmitoleic acid, oleic acid, and palmitoleic acid to palmitic acid (16:0) ratio were significantly increased in NAFLD across multiple lipid classes in agreement with the accelerated *de novo* lipogenesis syndrome. Linoleic acid (18:2ω6) was decreased (*p* < 0.05), with a concomitant increase in gamma-linolenic (18:3 ω6) and dihomo gamma-linolenic (20:3ω6) acids in both NAFL and NASH (P < 0.001 for most lipid classes). These changes are explained as an acceleration of essential fatty acids interconversion to compensate for the shortage in linoleic acid supply. The docosahexanoic acid (22:6 ω3) to docosapentenoic acid (22:5ω3) ratio was significantly decreased within phosphatidylcholine, and phosphatidylethanolamine pools, which was most marked in NASH subjects (P < 0.01 for phosphatidylcholine and P < 0.001 for phosphatidylethanolamine). The total plasmalogen levels were significantly decreased in NASH compared with controls (P < 0.05). The last two abnormalities indicate a slowdown of peroxisome activity as an index of severity in NASH. A stepwise increase in lipoxygenase metabolites 5(*S*)-hydroxyeicosatetraenoic acid (5-HETE), 8-HETE, and 15-HETE also accompanies the progression from normal to NAFLD to NASH. The oxidized byproducts of arachidonic acid parallel the inflammation reaction taking place. It may be concluded that although increased lipogenesis, desaturases, and lipoxygenase activities characterize NAFL and NASH, impaired metabolism of peroxisomal polyunsaturated fatty acids and non-enzymatic oxidation is associated with progression to NASH.

## 8. Lipidomic Application in Polycystic Ovarian Syndrome

By way of an example of the current gap existing between lipidomics capability and the listing of routine clinical and biochemical parameters prescribed for a particular pathology we can cite polycystic ovarian syndrome [36,37]. Pharmacological modifiers of the lipid metabolism including simvastatin and metformin have a profound effect on the clinical parameters of the disease and yet one is amazed by the limited application of lipidomics in their investigation. This illustrates the fact that only routine and global lipid assays are used and how the potential of lipidomics has yet to be exploited in a clinical trial conducted using lipid modifiers. Hormone levels were measured including total and free testosterone, LH, FSH, prolactin, SHBG (sex hormone binding globulin), and dehydroepiandrosterone sulfate (DHEAS) by specific assays. Insulin was assayed and insulin sensitivity index calculated using glucose and insulin levels obtained during an oral glucose tolerance test. Of the lipids only total cholesterol and triglycerides were determined by enzymatic colorimetric assays. Although significant benefits were observed in treatment with lipid modifying drugs the manifestation in terms of how the lipidome was altered and how this was ameliorating the condition remains unresolved and ripe for detailed lipidomic investigation.

## 9. Conclusions

The practical aspects of performing a lipidomic analysis has been reviewed elsewhere [38,39] and in this section we will concentrate on data reduction, analysis and presentation. These operations are crucial for correct interpretation of the results and to convey the results to the clinician in a meaningful and practical way. Multivariate analysis is required to extract the most significant lipid biomarkers from the large data files produced by MS-based lipidomics. Indeed, there is a major advantage in non-targeted lipidomic studies to report the vast array of lipids which can be modified under pathological and physiological conditions. The major advantage is that processing comes after recording and the possible multiple changes under these conditions can be illustrated meaningfully. The influences are composite (for example, inflammation, arachidonic and DHA-containing lipid oxidation, accelerated *de novo* lipogenesis, decreased ether-lipid synthesis and very long fatty acid metabolism, phospholipases and sphingomyelinases product metabolites can all participate in the final image given in the comprehensive lipidome analysis. An example of unsuspected interactions were evidenced after treatments combining exercise, essential ω-3 and ω-6 fatty acids in realistic animal models [40]. In man the combination with exercise also reveals unsuspected and occasionally disappointing results on the basis of gene studies [41] indicating that despite their effects on transcription ω-3 fatty acids were not effective as an adjunct for weight loss by modification of lifestyle and exercise in an overweight population. For all these reasons and before a few biomarkers consistent with particular lipid metabolism indicators are firmly established, the methods developed for multivariate analysis are expected to play a prominent role in human lipidomics. The methods are helpful to decipher pathological variations where the highly variable dietary influx of lipids can obscure the pathological features.

**Figure 4:**
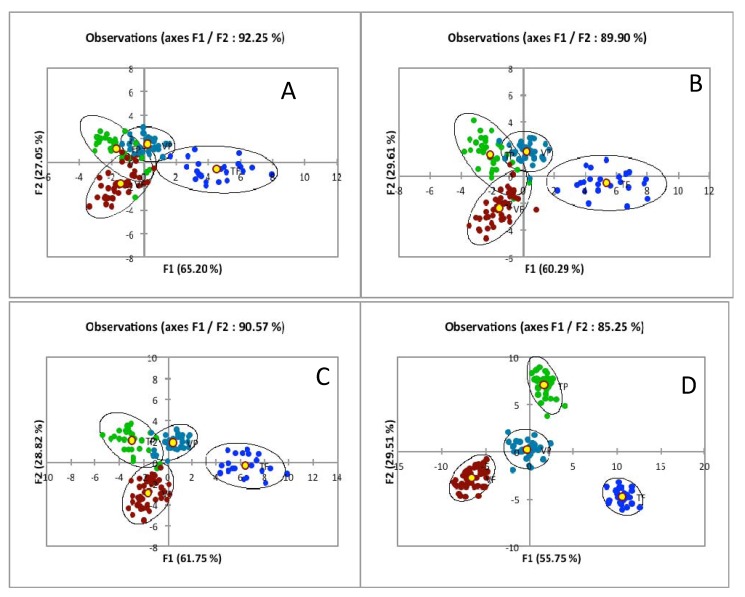
Discriminant Analysis of Liver Triglycerides in mice models prepared for Non-alcoholic fatty liver disease (NAFLD) treatment tests. Four different subgroups (n = 133) were identified with 2 genetic backgrounds (blue/green and dark blue/black symbols) as a function of the enriched or depleted lipid diets (green/dark blue and blue/black symbols). Triglyceride molecular species in the liver lipid extract were assayed by LCMS2. The multivariate analysis was conducted taking into account an increasing number of molecular species A = 11, B = 21, C = 31 and D = 51. In each subgroup the centroid (yellow dot) and confidence ellipses are calculated and positioned to maximize the discrimination of subgroups in the factor plan F1/F2. It is apparent that the within-class dispersion is decreased but the separation (between-classes covariance) is increased between centroid subgroups as a function of the number of molecular species which is taken into account for calculation. However the confusion matrix reveals that the rate of erroneous assignment to subgroups of test samples is sharply rising with the number of molecular species taken into account in the calculation (A, 0%; B, 0%; C, 77%; D, 83%). See Section 5.

By the same token, the multivariate analysis informs on the consistency of large collections of lipidomics data. Figure 4 shows the result of such a discriminant factor analysis of liver triglyceride molecular species. The study was aimed to select a realistic and sensitive animal model to be used for NAFLD drug testing. The recordings show more than 200 molecular species detected. Indeed, the differences revealed by the profiles between the 2 genetic backgrounds established by back-crossing of the C57Bl/6 strain after transgenesis have to be deciphered and consolidated as a reliable index before the drug testing is started. The discriminant analysis is helpful to select a combination of biomarkers maximizing the discrimination of the subgroups. As shown in Figure 4 an increasing number of triglyceride species are entered in the calculation. Because species are ranked by their abundance, the higher the number of species, the higher the noise contaminating the meaningful signals in the mass spectrum. As a result the discrimination between subgroups is increased with more numerous mass peaks entering the statistics but the number of erroneous subgroup assignments of observations makes the classification meaningless. Indeed the sensitivity of detection of a particular MS spectrometer and the available lipid amounts in the extracts change the threshold number of molecular species which can be usefully assayed.

Development of routine methods of lipidomic analysis for clinical and biomedicine requires automated equipment and a well found basis for linking particular features of the lipidome to specific pathologies. The development of the necessary hardware is now equal to the challenge. Further work is required in two areas. Firstly, the underpinning science linking lipid biomarkers to clinical conditions is still largely unexplored. Secondly, data processing and presentation needs to be improved so that interpretation and action can be translated into a clinical setting. The future holds great promise for therapies and treatments for afflictions that beset our growing populations.
